# Effectiveness of acupuncture, special dressings and simple, low-adherence dressings for healing venous leg ulcers in primary healthcare: study protocol for a cluster-randomized open-labeled trial

**DOI:** 10.1186/1472-6882-8-29

**Published:** 2008-06-11

**Authors:** Jorge Vas, Manuela Modesto, Camila Mendez, Emilio Perea-Milla, Inmaculada Aguilar, Jesus Manuel Carrasco-Lozano, Vicente Faus, Francisco Martos

**Affiliations:** 1 Pain Treatment Unit, Primary Healthcare Centre, Dos Hermanas, Spain; 2 Andalusian Public Health System, Sevilla, Spain; 3 Support Research Unit (Network and Cooperative Research Centres of Epidemiology. CIBERESP), Costa del Sol Hospital, Marbella, Spain; 4 Costa del Sol Hospital, Marbella, Spain; 5 Department of Pharmacology, Malaga University, Spain

## Abstract

**Background:**

Venous leg ulcers constitute a chronic recurring complaint that affects 1.0–1.3% of the adult population at some time in life, and which corresponds to approximately 75% of all chronic ulcers of the leg. Multilayer compression bandaging is, at present, the only treatment that has been proved to be effective in treating this type of ulcer. There is no consensus, however, about the dressings that may be applied, beneath the compression, to promote the healing of this type of ulcer, as there does not seem to be any added benefit from using special dressings rather than simple, low-adherence ones. As well as analgesia, acupuncture provokes peripheral vasodilation, in skin and muscles – which has been demonstrated both experimentally and in clinical practice – probably due to the axon reflex, among other mechanisms. The aim of the present study is to measure the effectiveness and cost of compression treatment for venous leg ulcers combined with special dressings, in comparison with low-adherence ones and acupuncture.

**Methods/design:**

Cluster-randomized open-labeled trial, at 15 primary healthcare clinics in the Sevilla-Sur Healthcare District, with a control group treated with compression bandaging and low-adherence dressings; the experiment will consist, on the one hand, of the compression treatment applied in combination with special dressings (Treatment 1), and on the other, the compression treatment applied in association with low-adherence dressings, together with acupuncture (Treatment 2).

**Discussion:**

The results will be measured and recorded in terms of the median time elapsed until complete healing of the ulcer, and the rate of complete healing at 3 months after beginning the treatment. An economic analysis will also be made.

This study, carried out in the context of real clinical practice, will provide information for decision-taking concerning the effectiveness of special dressings. Moreover, for the first time a high-quality study will evaluate the effectiveness of acupuncture in the process of healing venous leg ulcers.

**Trial registration:**

Current Controlled Trials ISRCTN26438275.

## Background

Venous leg ulcers (VLU) constitute a chronic recurring complaint that affects 1.0–1.3% of the adult population at some time in life, and which corresponds to approximately 75% of all chronic ulcers of the leg [1]. They are caused by sustained venous hypertension, which is the result, in almost 50% of cases, from superficial venous insufficiency or from malfunctioning valves in perforating veins, with a normally-functioning deep venous system. Other factors that may result in sustained venous hypertension are those that impede the pumping function of the leg muscles, such as conditions that reduce spontaneous movements (Parkinson's disease, stroke, spinal cord injuries or excessive sedation). This type of chronic venous insufficiency is characterized by oedema, venous dilation, painful legs and stasis dermatitis. VLU patients have a worse quality of life than non-affected persons of the same age due to the pain, malodour and loss of independence that it causes [2]. The precise procedure by which hypertension leads to ulceration has yet to be clarified, but various mechanisms are known to be capable of intervening in its development and maintenance, such as plasma extravasation, pericapillary deposits of fibrin [3], disorders of the fibrinolytic system [4], the fixing or retention of growth factors by macromolecules in the dermis, or leukocytes in the veins of the legs [5-7].

In the USA, VLUs have been estimated to cost a billion dollars a year, representing an average cost of over 40,000 dollars during the lifetime of each patient [1], without taking into account the financial effects of impaired quality of life, sick leave and frequent hospitalization. In the United Kingdom, the average cost of treating venous ulcers varied between 814 to 1,994 euros per year, of which a large proportion is devoted to nursing expenses [8,9]. In Europe, the total cost of VLU treatment amounts to 1% of the annual healthcare budget [10], with an estimated mean cost of 250 euros in Spain per patient, per year of treatment [11].

Developing a healthcare plan for VLU patients includes curing active ulcers and preventing relapses. To achieve these goals, such a plan must address the treatment of infections associated with these ulcers, preventing the development of infection, stimulating granulation and epithelial tissues, compression, raising the leg position, improving mobility, reducing obesity, improving nutrition and, in some cases, performing surgery [12,13].

Perhaps the most controversial aspect of the local treatment of VLUs concerns the type of dressings to use [14]. The use of wet dressings has been shown to favour granulation and epithelialization processes and, therefore, the healing of the ulcer, but no evidence has been provided to assist in choosing between special dressings and simple, low-adherence ones [15]. The application of external compression causes various complex physiological and biochemical effects that affect the venous, arterial and lymphatic systems. Provided the degree of compression does not adversely affect arterial blood flow, and that the correct techniques and materials are applied, the effects of compression may be very positive, reducing oedema and pain, whilst at the same time favouring the healing of ulcers caused by venous insufficiency. However, there are many ways in which this compression may be applied, and there is no consensus as to which is best [16]. Depending on the size of the ulcer, healing rates with compression treatment range from 40–70% at three months to 50–80% at 6 months [17,18]. The latest systematic review carried out by the Cochrane Collaboration recommends compression therapy and low-cost, low-adherence dressings as a first-line treatment for healing VLUs [15].

In addition to analgesia, acupuncture provokes peripheral vasodilation in the skin and in muscles, probably due to the axon reflex [19-21]. Stimulation of Aδ and C fibres releases vasoactive neuropeptides, as well as proinflammatory agents like the calcitonin gene? related peptide (CGRP), the P substance (SP), neurokinin A (NKA), opioids, galanin, somatostatin and vasoactive intestinal peptide (VIP) [22,23]. Deep, prolonged vasodilation may also be mediated by CGRP [24-26]. Some studies have suggested that the neuropeptides released by sensory nerve stimulation are beneficial in maintaining skin integrity [23,27,28], for healing ulcers [29,30] and for peripheral vascular disorders [31,32]. Another key element in regulating the vascular tone and the blood flow is the endothelial synthase of nitric oxide (NO), which is responsible for catalyzing the NO [33], which is active in arteriolar vasodilation and reduces peripheral resistance, facilitating normal blood flow to the tissues [34]. It has been shown that the expression of NO-synthase (NOS) is greater in skin regions where acupuncture points and channels are located. In these areas, moreover, there are raised levels of NO in the blood after acupuncture [35,36], which suggests that the acupunctural stimulation of the sensorial nerves may act as an *in vivo *modulator of NO levels [32]. Although studies have been carried out to examine the effectiveness of acupuncture in treating VLUs, these have been of poor quality and have reported contradictory results [37-39].

In this paper, we propose a multicentre cluster-randomized study, with a predetermined sample size, and appropriate follow up, assessing the patients' quality of life and employing an objective results measure that will enable us to accurately evaluate the length of time needed for patients' ulcers to heal, thus meeting the recommendations of the Cochrane Collaboration's review body [15,16].

## Methods/Design

### Research aims and questions

The aim of this study is to determine whether the combination of compression bandaging with simple, low-adherence dressings and acupuncture is more effective than when the same bandaging is employed in combination with special dressings or with simple, low-adherence dressings, but no sensory stimulation (acupuncture), with respect to the complete healing of venous leg ulcers.

The following research questions are proposed:

▪ In terms of the healings of venous leg ulcers, is it better to employ compression bandaging associated with sensory stimulation (acupuncture) and simple dressings, than the same bandaging without sensory stimulation, with special dressings, or than the compression bandaging without sensory stimulation and with simple dressings?

▪ What are the differences between healing treatments with special dressings and those with simple, low-adherence ones, in terms of the complete healing of venous leg ulcers?

▪ How long does it take for venous leg ulcers to recur?

▪ How effective is the treatment in terms of reducing pain intensity?

▪ How effective is the treatment in terms of reducing the amount of analgesic and anti-inflammatory medicines consumed?

▪ Are there any quality of life changes related to the health of patients with venous leg ulcers?

▪ What is the total financial cost of each of the treatments studied?

▪ What is the cost-effectiveness ratio of the treatments studied, for patients with venous leg ulcers?

▪ What is the opportunity cost of treatments not implemented?

### Design

Open-labeled, controlled multicentre prospective study, with random cluster allocation to treatment groups (Primary Healthcare Clinics) in the Sevilla-Sur Health District (Andalusian Public Health System).

### Study duration

April 2008-December 2010

### Subjects

#### Target population

patients with venous leg ulcers who request treatment at the Primary Healthcare Clinic.

#### Study population

patients with venous leg ulcers who request treatment by a healthcare professional (doctor or nurse) at one of the Primary Healthcare Clinics in the Sevilla-Sur Health District taking part in the study, with at least one active venous leg ulcer (open, CEAP C6 [40] with a diameter exceeding 1 cm.

Patients with one or more of the following will be **excluded **from the study:

▪ Arterial pathology (systolic pressure in the ankle of less than 80 mm Hg or ankle/arm systolic index less than 0.8).

▪ Diabetic ulcer of the foot, rheumatoid arthritis or systemic vasculitis.

▪ Use of anticoagulants

▪ Pregnancy

#### Sampling units

the Primary Healthcare Clinics participating in the study.

#### Analysis units

per Healthcare Clinic (according to the group assigned) and per patient.

#### Randomization and blinding procedure

a list of random numbers will be created, using statistical software, to which the researchers involved in the study will not have access. This list will be applied to the list of participating Clinics until the clusters for each group are obtained. Three types of cluster will be used, according to the treatment for the venous leg ulcer provided at the Clinic (**A: **compression therapy plus simple, low-adherence dressings and acupuncture; **B: **compression therapy plus special dressings; **C: **compression therapy plus simple, low-adherence dressings). Before beginning, the list of Clinics will be randomly ordered, by drawing lots blindly, with no repetitions, from a bag containing the names of all the Clinics taking part. None of those participating in this process will belong to the research group, and none of them will play any further part in the study.

### Ethical criteria

The ethical validity of this study has been analyzed and approved by the Andalusian Government Committee for Clinical Trials, following the approval of the corresponding Research Commission at each of the participating Clinics. The study design takes into account the fundamental principles set out in the Helsinki Declaration, and those of the Council of Europe Convention concerning human rights and biomedicine, as well as the requirements under Spanish law in the field of biomedical research, the protection of personal data, and bioethics. All the patients involved must sign their informed consent to the proposed clinical research procedures. During the course of the study, audits will be performed as required by the relevant Research and Ethics Committee, as well as those of each Clinic's Quality Committee, independently of any external audits (such as that of the research financing body) that may be necessary.

### Sample size

The sample size has been calculated following the parameters of Campbell et al. [41], on the basis of historical data [18]. The success rate for complete healing of venous leg ulcers at 3 months, with compression bandaging plus special dressings, is 44%. With an alpha (significance) level of 0.05 and a power of detection of differences of 80%, assuming a two-tailed approach, the total sample size needed will be 375 patients, in order to detect differences of 15% between the groups, as regards the rate of complete healing of venous leg ulcers at 3 months. This calculation assumes a possible dropout rate of 5% and an intra-cluster coefficient of correlation of 0.05 (according to the expected variance of the response within a single cluster). There will be 15 clusters (Primary Healthcare Clinics) and so each Clinic will need to recruit 25 subjects. Therefore, 3 treatment arms will be formed, with 125 patients in each (with 5 Clinics per arm).

### Interventions

**Figure 1:**
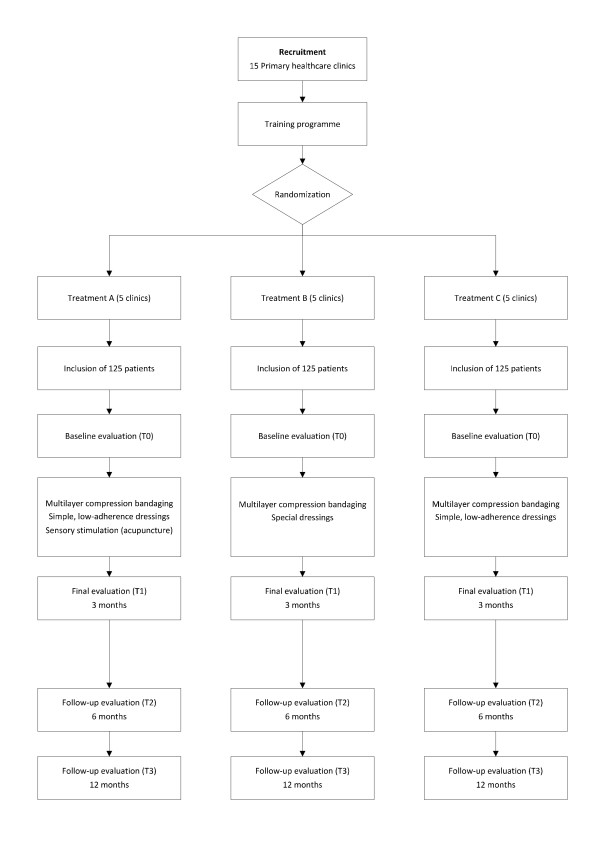
**Flow diagram for the study**. Flow diagram for the diverse phases of the study.

The compression treatment and application of dressings will be performed in accordance with the standard procedure and the criteria of the nursing staff responsible. A priori, one treatment session per week is planned, but this may be modified in the light of the patient's response. Follow up of the patients recruited into this study will be continued for one year after the randomization process. The acupuncture sessions in Group A will be given at the same time as the cleansing of the ulcer and the changing of the compression bandage, and will continue for 3 months, or until the ulcer has healed completely (whichever occurs first). If, after these 3 months, the ulcer has not healed, the sessions will be continued (as before, together with the cleansing of the ulcer) for another 3 months. The acupuncture treatment will not be continued for longer than 6 months (figure 1).

The patients taking part in the study will be given recommendations about suitable hygiene-posture routines (resting with the legs raised, not standing for long periods, taking regular walks, keeping the skin well hydrated) and nutritional advice, if necessary. Both the compression treatment and the application of dressings will continue until the ulcer has completely healed. If a recurrence occurs before the one-year follow-up period has ended, the patient will be entered into a sub-cohort and given a new treatment plan.

#### Compression therapy (Groups A, B and C in the study)

Compression therapy will be given to all the participants in the study. Once the patient has been examined and the ankle/arm index calculated by means of a Doppler scan, an evaluation will be made of the state of the skin and of the shape of the leg, after confirming the absence of neuropathology, arterial pathology and cardiac insufficiency. The bandaging will be of a Class 3, high-compression multilayer type [42], following cleansing and application of the dressing, as follows: 1) With a 50% overlap, apply a cotton bandage in a spiral pattern from the base of the toes to just below the knee, without imposing any tension; 2) Apply an elastic bandage in a spiral pattern, with 50% overlap and stretched to 50% of its extension, from the base of the toes to just below the knee; 3) Apply a cohesive elastic bandage, with 50% overlap and stretched to 50% of its extension, from the base of the toes to just below the knee, and apply light pressure to ensure the adherence of the bandage. The oedema of the leg should be reduced by raising the limb for 15 minutes before applying compression.

#### Treatment of the ulcer for the patients given low-adherence, simple dressings (Study groups A and C)

▪ Cleaning: with tap water and mild soap [43-45], followed by careful drying

▪ Debridement: if necrotic tissue or sloughing are observed, perform enzymatic debridement, which may be combined with excision debridement, if necessary

▪ Managing infection: if infection is suspected, take a sample and begin treatment with silver sulfadiazine for a maximum of 2 weeks. If a culture study confirms the infection, treat using oral antibiotics

▪ Dressings: simple, low-adherence type (paraffin-impregnated tulle gras)

Any adverse reaction or side effect that may occur should be recorded in the Data Record (DR), with a detailed description and the date of occurrence.

#### Treatment of the ulcer for the patients given special dressings (Study group B)

▪ Cleaning: with tap water and mild soap, followed by careful drying

▪ Debridement: if necrotic tissue or sloughing are observed, perform enzymatic debridement, which may be combined with hydrogel to favour autolysis. If necessary, excision debridement should be applied

▪ Managing infection: if infection is suspected, take a sample and begin treatment with silver dressings (hydrofibre or hydropolymer) for a maximum of 2 weeks. If a culture study confirms the infection, treat using oral antibiotics

▪ Dressings: Cover with hydropolymers. Use alginate or hydrofibre dressings if exudate must be controlled

Any adverse reaction or side effect that may occur should be recorded in the DR, with a detailed description and the date of occurrence.

#### Acupuncture (Study Group A)

The nursing staff will be trained in the specific techniques of localization, puncture and manipulation in order to apply acupuncture at the perilesional zone of the ulcer(s), in healthy skin, using 4–8 needles, and at 2 points located on either side of the tibial crest of the affected limb (*Yinlingquan *SP9 and *Zusanli *ST36). The puncture will be carried out after cleansing of the ulcer. The needle to be inserted is a sterile, single-use filiform acupuncture needle, 30 mm long and 0.30 mm in diameter, fitted with a guide tube (S-J3030, manufactured by Seirin Corporation). Application will be made at each of the above-mentioned points, after sterilizing the skin, and with the patient lying face up or sideways. The puncture will be made vertically, unless otherwise specified, to a depth of 10–28 mm. The insertion will be followed by broad bidirectional rotational movements of the needle handle in order to produce the sensation of *Deqi*, commonly described as an irradiated feeling. The needle will be held in place for 20 minutes, and moved for 10 seconds every 5 minutes (4 manipulations per session). The acupuncture sessions will be applied for the first 3 months of this treatment programme or until the ulcer has completely healed. If complete healing is not achieved in this period, the sessions may be prolonged for another 3 months, after which the acupuncture treatment will be discontinued.

For each session, the number of needles used and the exact location of their application will be recorded. Any adverse reaction or side effect arising from this treatment should be recorded in the DR, with a detailed description and the date of occurrence.

A priori, the ulcer should be cleansed once weekly, the same frequency as the sensory stimulation with acupuncture. Nevertheless, this may be increased or decreased, at the discretion of the nursing staff.

All treatment will be given by the professional healthcare staff (nurses) employed at the Primary Healthcare Clinics, coordinated by the treatment programme head in each case. In order to standardize criteria and training procedures in the use of compression bandages, the data record system and the application of the study treatments, a **specific ad-hoc training course **will be given to all the healthcare staff involved in the study. This training will be structured as follows:

▪ Aims: acquisition of skills in applying acupuncture protocols for venous ulcers, in applying treatment techniques using the different kinds of dressing and in using the data record system. Standardization of criteria for applying compression bandages.

▪ Content: differential diagnosis of leg ulcers, calculation of the ankle/arm index, selection and localization of acupuncture points, the insertion of needles, the preparation and application of ulcer cleansing treatment, the reaction to possible infection, training in techniques with compression bandages, giving instructions to patients, implementing follow up, and giving all the study participants hygiene, posture and nutrition recommendations

▪ Instruction method: workshop

▪ Duration: 10 teaching hours

### Outcome measures

#### Primary outcome

##### Time until complete healing is achieved

Complete healing is defined as the complete epithelialization of all the leg venous ulcers, not just the reference one.

#### Secondary outcome variables

##### Complete healing at 3 months after beginning treatment

Complete healing is defined as the complete epithelialization of all the leg venous ulcers, not just the reference one (table 1).

**Table 1:** Outcome measures. Work scheme with description of assessment visits and times.

	**Baseline (T0)**	**Weekly treatment**	**Evaluation at 3 months (T1)**	**Weekly treatment (if necessary)**	**Evaluation at 6 months (T2)**	**Evaluation at 12 months (T3)**
Sociodemographic data	X					
Ankle/arm index	X					
Nutritional study	X					
Localization of ulcer(s)	X		X		X	X
Area of main ulcer	X	X	X	X	X	X
Duration of the main ulcer	X					
Exudate culture (if necessary)	X	X	X	X	X	X
Changes in the size of the main ulcer		X	X	X	X	X
Characteristics of the main ulcer	X	X	X	X	X	X
Complete healing (date)		X	X	X	X	X
Pain intensity		X	X	X	X	X
SF-12	X		X		X	X
Adverse events/effects		X	X	X	X	X
Compliance with hygiene-posture recommendations	X		X		X	X
Location of treatment (clinic/home)		X		X		
Time employed in treatment		X		X		
Materials employed in treatment		X		X		
Number of needles used(Group A)		X		X		
Obtention of Deqi (Group A)		X		X		

##### Changes in the size of the surface area of the ulcer (in cm^2^)

When the baseline evaluation is made, the ulcer will be measured; if there are more than one, the largest ("the main ulcer") will be assessed. Nevertheless, the outcome variable of complete healing will be determined on the basis of all the ulcers observed on the patient when the baseline assessment is made. The area of the ulcer will be calculated using a compact digital tablet (Visitrak), with which the area can be assessed quickly, conveniently and accurately. The outline of the ulcer is drawn on a tracing sheet used as the digital screen; this has three layers to minimize the risk of cross contamination or secondary infection, and the layer in contact with the patient is sterile. The surface on which the data are recorded is kept clean and may be included in the patient's case history.

##### Pain intensity measured on a visual analogue scale

There exists sufficient evidence to corroborate the validity of the visual analogue scale of pain intensity. Many studies have demonstrated the validity of the concept [46] and its reliability [47,48]. With this method, the subjective intensity of pain may be measured quickly and straightforwardly. The patient is asked to mark the degree of pain intensity on a millimetric scale, from 0 (no pain) to 100 (the worst pain imaginable).

**Changes in the health-related quality of life **at 3, 6 and 12 months (12-item Short Form health survey, version 2) [49].

##### Adverse events – Adverse effects

A record will be kept of any adverse events or adverse effect observed by healthcare staff or reported by the patient or by his/her carer.

##### Cost-utility

Health utility will be measured in terms of the quality-adjusted life years (QALY) gained for each patient, calculated from the area beneath the SF-12 curve at 3 months from the start of treatment.

##### Opportunity cost

this will include the sum of the outputs from the activities potentially left undone as a result of providing a given treatment, rather than a possible alternative, together with the potential financial cost of the resources employed. Using the ABC method, the resources imputed to each patient will be distributed in a multidisciplinary table based on a modular "counter"-type framework for each case, in order to apply the analytical model and to obtain the SF-12 curve showing the variation in health-related quality of life and the potential cost savings for the Public Healthcare System.

#### Covariables

The covariables recorded will be the patient's age, sex, educational level, occupation, income, weight (kg), height (cm), mobility, systolic arterial pressure, diastolic arterial pressure, arm/ankle index and ankle diameter (cm), the duration of the active ulcer (weeks), the location of the ulcer (if more than one ulcer is present, stating the location of the largest one, which is taken as the reference ulcer), whether the ulcer is unilateral or bilateral, the number of ulcers on the leg(s), whether the ulcer is new or recurrent (in the latter case, recording the time since the first appearance, in years and months), the signs of granulation at the base of the ulcer, the signs of epithelialization at the edges of the ulcer, the presence of fibrinous and/or necrotic tissue, the presence of lipodermatosclerosis, and the presence of infection. Other variables recorded will include dependence on tobacco, alcohol or other psychoactive substances, the presence of diabetes mellitus, the results of a nutritional study (haemogram and biochemical tests: albumin, prealbumin, transferrin and proteinogram), the time elapsed until recurrence occurs (weeks), compliance with hygiene-posture recommendations, self care, and the assistance or otherwise of non-professional carers. In addition, a record will be kept of all medication taken (corticoids, immunosuppressors, anticoagulants, etc.) and information about possible cultures of the ulcer exudate.

### Data handling

The data on the variables of interest will be recorded on a purpose-designed form to be completed by each researcher at each clinic. This information will be entered into a database for subsequent statistical analysis.

### Data collection

The routine follow-up evaluation, to be made following cleansing session, will be performed by the nursing staff responsible. The baseline evaluation (T0), that of the results at 3 months (T1), and the follow-up evaluations at 6 and 12 months (T2 and T3) will be carried out by the healthcare personnel responsible for the treatment programme (and who will be blinded with respect to the group to which each patient is assigned).

### Measurements (measurement times)

#### Baseline evaluation basal (T0)

▪ The data obtained in the examination made prior to beginning treatment will include personal and sociodemographic characteristics, background and case history, ankle/arm index, nutritional status, previous treatment received, results of the ulcer exudate culture, the dimensions of the ulcer and observations regarding its condition, location and duration in an active state, as well as about the patient's health-related quality of life.

▪ Follow up. At each visit, the nurse applying the treatment will record the macroscopic status of the ulcer, any adverse events that may have occurred, the time employed in providing the treatment, the location of the treatment (clinic/home) and the name of the person performing it. If total healing of the ulcer is detected during a routine examination, the treatment programme official at the relevant Clinic should be informed. For the patients in Group A, a record should also be kept of the data concerning the acupuncture session (the number and location of local points).

#### Follow-up evaluation at 3 months after starting treatment (T1)

At three months after starting the treatment, a fresh assessment will be made of the patient's condition, quality of life and compliance with hygiene-posture recommendations, as well as of the physical status of the ulcer (appearance and size).

#### Follow-up evaluation at 6 months after starting treatment (T2)

With the same content as the previous evaluation.

#### Follow-up evaluation at 12 months after starting treatment (T3)

With the same content as the previous evaluation.

### Data analysis

The data analysis will be carried out by personnel blinded to the treatment groups, and for two types of populations: (1) intention to treat (ITT), with all the patients randomized; (2) per protocol (PP), including only the patients with no more than slight variances from the protocol.

The baseline variables for the different groups will be compared, to test the homogeneity produced by random allocation, in terms of differences of the means and of proportions, and the only differences taken into consideration in this comparison will be those that are clinically relevant for subsequent analysis. The magnitude of the difference in any imbalance produced by the random allocation to groups will be evaluated by using ratios of the means, or medians, or proportions, bearing in mind whether the level of analysis is per cluster or per individual.

The final analysis will be carried out as a univariate survival analysis (using the Kaplan-Meyer method and the log-rank-test for inter-group comparison). The hazard ratios for each treatment will be calculated, together with the corresponding NNTs, calculating a 95% confidence interval, using Cox's proportional risk regression model. The assumption of proportionality will be assessed from the interaction between the time elapsed until success (total healing) and treatment group, and will be tested graphically by observing the parallelism in the log-log curves. A multilevel analysis will be carried out to adjust for possible unbalanced variables in the baseline analysis, taking the patients as level 1 and the Primary Healthcare Clinics as level 2. The interactions between clinic and treatment group will be examined, as will the possible variability in the intercept. The level of significance will be set at p < 0.05, for all the tests made. Cost-effectiveness, cost-utility and opportunity cost analyses will be made.

## Discussion

We have identified three shortcomings in the present study design: the first arises from its conceptualization, in attempting to measure the effectiveness of procedures (acupuncture) whose efficacy has yet to be proven. It might be argued that a previous step should be to design a double-blinded study of effectiveness, comparing the above methods with a placebo procedure. However, apart from the impossibility of implementing a double-blind test for acupuncture, our viewpoint is to seek to determine the validity of clinical practices that are feasible in pragmatic contexts. The second limitation concerns the variability of the effects produced by nursing care; nevertheless, the cluster design employed is aimed at minimizing the risk of habit-formed contamination in the treatments applied and that of contamination between patients, in the sense of their sharing information about the results. These problems are addressed by standardizing the protocols/procedures and by organizing meetings to standardize the criteria applied, as well as by performing an a posteriori statistical analysis of the influence of the "therapist" factor. Finally, the fact that the outcome variable assessed is that of the complete healing of all leg venous ulcers suffered by the patient complicates analysis, but we believe a more limited study would have less probability of being of interest to patients concerned with the time elapsed until the complete healing of all their ulcers.

## Competing interests

The authors declare that they have no competing interests.

## Authors' contributions

JV conceived the study, designed the study protocol, sought funding and ethical approval and wrote the manuscript. MM contributed substantially to designing the ulcer-treatment procedure. All authors contributed to the research design, read, made critical revisions and approved the final manuscript.

## Pre-publication history

The pre-publication history for this paper can be accessed here:


